# Host–Receptor Post-Translational Modifications Refine Staphylococcal Leukocidin Cytotoxicity

**DOI:** 10.3390/toxins12020106

**Published:** 2020-02-06

**Authors:** Angelino T. Tromp, Michiel Van Gent, Joris P. Jansen, Lisette M. Scheepmaker, Anneroos Velthuizen, Carla J.C. De Haas, Kok P.M. Van Kessel, Bart W. Bardoel, Michael Boettcher, Michael T. McManus, Jos A.G. Van Strijp, Robert Jan Lebbink, Pieter-Jan A. Haas, András N. Spaan

**Affiliations:** 1 Department of Medical Microbiology, University Medical Center Utrecht, 3584 CX Utrecht, The Netherlands; 2 Department of Microbiology, University of Chicago, Chicago, IL 60637, USA; 3 Department of Microbiology and Immunology, UCSF Diabetes Center, Keck Center for Noncoding RNA, University of California, San Francisco, San Francisco, CA 94143, USA; 4 Medical Faculty, Martin Luther University Halle-Wittenberg, 06120 Halle (Saale), Germany; 5 St. Giles Laboratory of Human Genetics of Infectious Diseases, Rockefeller Branch, The Rockefeller University, New York, NY 10065, USA

**Keywords:** *Staphylococcus aureus*, bi-component pore-forming toxins, leukocidins, receptors, G-protein coupled receptors, post-translational modifications

## Abstract

Staphylococcal bi-component pore-forming toxins, also known as leukocidins, target and lyse human phagocytes in a receptor-dependent manner. S-components of the leukocidins Panton-Valentine leukocidin (PVL), γ-haemolysin AB (HlgAB) and CB (HlgCB), and leukocidin ED (LukED) specifically employ receptors that belong to the class of G-protein coupled receptors (GPCRs). Although these receptors share a common structural architecture, little is known about the conserved characteristics of the interaction between leukocidins and GPCRs. In this study, we investigated host cellular pathways contributing to susceptibility towards *S. aureus* leukocidin cytotoxicity. We performed a genome-wide CRISPR/Cas9 library screen for toxin-resistance in U937 cells sensitized to leukocidins by ectopic expression of different GPCRs. Our screen identifies post-translational modification (PTM) pathways involved in the sulfation and sialylation of the leukocidin-receptors. Subsequent validation experiments show differences in the impact of PTM moieties on leukocidin toxicity, highlighting an additional layer of refinement and divergence in the staphylococcal host-pathogen interface. Leukocidin receptors may serve as targets for anti-staphylococcal interventions and understanding toxin-receptor interactions will facilitate the development of innovative therapeutics. Variations in the genes encoding PTM pathways could provide insight into observed differences in susceptibility of humans to infections with *S. aureus*.

## 1. Introduction

*Staphylococcus aureus* is a Gram-positive bacterium that colonizes the skin and anterior nares of 20%–30% of the general human population [1]. *S. aureus* also causes a variety of diseases, ranging from superficial skin and soft tissue infections to severe invasive infections with a poor prognosis and high mortality [2]. Upon infection, *S. aureus* is faced with the host humoral and cellular innate immune response [3]. *S. aureus*, in return, secretes an arsenal of virulence factors to circumvent host defenses and to avoid killing by phagocytes [4]. An important group of *S. aureus* virulence factors, the leukocidins, specifically target and lyse host phagocytes [5,6]. *S. aureus* leukocidins are bi-component beta-barrel pore-forming toxins [6]. Human *S. aureus* isolates secrete up to five leukocidins: Panton-Valentine leukocidin (PVL), γ-haemolysin AB (HlgAB) and CB (HlgCB), leukocidin ED (LukED) and leukocidin AB (LukAB, also knowns as LukGH) [6]. Based on chromatography elution profiles, the two individual leukocidin subunits are designated S- (slow migrating) or F- (fast migrating) components [5].

Proteinaceous targets have been identified for all *S. aureus* leukocidins. The S-component of the leukocidins, with the exception of LukAB, target specific G-protein coupled receptors (GPCRs) expressed on the surface of host cells [5]. The C5a anaphylatoxin chemotactic receptor 1 (C5aR1, also known as CD88) and C5a anaphylatoxin chemotactic receptor 2 (C5aR2, also known as C5L2) were identified as targets for PVL and HlgCB [7,8]. LukED targets leukocytes via CC-chemokine receptor 5 (CCR5), as well as CXC chemokine receptor 1 (CXCR1) and CXC chemokine receptor 2 (CXCR2) [9,10]. HlgAB targets CXCR1, CXCR2 and CC-chemokine receptor 2 (CCR2) [8]. In addition, HlgAB and LukED both target the Duffy antigen receptor for chemokines (DARC, also known as ACKR1), an atypical chemokine receptor expressed on erythrocytes [11]. Although these receptors share a seven-transmembrane spanning structural architecture common to all GPCRs, little is known about the conserved or divergent characteristics of the interaction between leukocidins and their respective GPCR host-counterparts. The apparent redundancy of the leukocidins in terms of overlapping receptors and host target cell populations remains enigmatic. Furthermore, additional molecular determinants of the host target cell involved in leukocidin-receptor interactions are incompletely understood.

In this study, we applied a genome-wide CRISPR/Cas9 library screen to identify host factors involved in PVL- and HlgCB-mediated cytotoxicity. We identify post-translational modification (PTM) pathways that refine GPCR-mediated susceptibility of human phagocytes to leukocidins. Sulfation-mediated receptor-employment serves as a major and conserved feature for C5aR1-interacting leukocidins. In contrast, sialylation rather than sulfation is a major PTM motif facilitating cytotoxicity of CXCR2-targeting leukocidins. These findings further substantiate the complexity underlying the divergent interaction between *S. aureus* bi-component pore-forming toxins and their target cells.

## 2. Results

### 2.1. PTM Pathways Affect Susceptibility to PVL and HlgCB Cytotoxicity.

**Figure 1:**
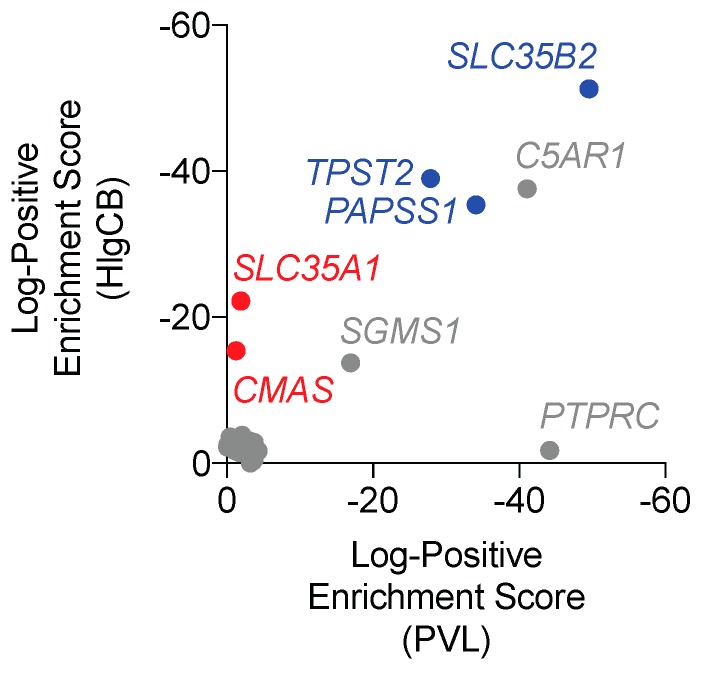
Genome-wide CRISPR/Cas9 library screen reveals post-translational modification pathways involved in PVL and HlgCB toxicity. Cellular factors involved in PVL- and HlgCB-mediated cytotoxicity as identified by the introduction of a genome-wide sgRNA library in U937-C5aR1-SpCas9 cells coupled to deep sequencing. Visualized are the most significantly enriched genes after PVL and HlgCB challenge as calculated by the MaGeCK ‘positive enrichment score’. See Tables S1 and S2 for the full list of genes. Grey: genes encoding known host cellular factors; blue: genes belonging to the tyrosine sulfation pathway; red: genes involved in the sialylation pathway.

To identify host factors involved in PVL- and HlgCB-mediated susceptibility of human phagocytes, a genome-wide CRISPR/Cas9 library screen for both PVL- and HlgCB resistance was set up in human U937 promyelocytic cells. Cells were sensitized to PVL- and HlgCB mediated pore-formation by overexpressing C5aR1 (U937-C5aR1), followed by the introduction of a human codon-optimized nuclear-localized *S. pyogenes* cas9 gene (U937-C5aR1-SpCas9). Host factors involved in PVL and HlgCB toxicity were detected via the introduction of a genome-wide sgRNA library coupled to deep sequencing, allowing for the identification of genes inactivated in cells surviving toxin treatment. *C5AR1*, encoding the LukS-PV and HlgC receptor C5aR1, was identified as a top hit in both the HlgCB- and PVL-resistance screen, validating the screening method (Figure 1 and Tables S1 and S2). As recently reported, *PTPRC*, encoding the Luk-F-PV specific F-component receptor CD45, was identified in the PVL-, but not HlgCB-screen [12]. Consistent with screenings using other pore-forming toxins, the gene encoding the alpha-hemolysin (Hla) determinant sphingomyelin synthase 1 (*SGMS1*) [13] was also identified for both PVL and HlgCB (Figure 1).

Unexpectedly, the screenings for both PVL and HlgCB-resistance revealed enrichment for the genes encoding the Solute Carrier Family 35 Member B2 (*SLC35B2*), 3′-Phosphoadenosine 5′-Phosphosulfate Synthase 1 (*PAPSS1*) and Tyrosylprotein Sulfotransferase 2 (*TPST2*) (Figure 1). In addition, the screening for HlgCB-resistance was enriched for the genes encoding the Solute Carrier Family 35 Member A1 (*SLC35A1*) and Cytidine Monophosphate N-Acetylneuraminic Acid Synthetase (*CMAS*) (Figure 1). SLC35b2, PAPSS1 and TPST2 are key components of the protein sulfation pathway in which tyrosine residues are enzymatically decorated with a sulfate group [14]. SLC35a1 and CMAS are part of the sialylation pathway, a modification process resulting in the attachment of sialic acids to other molecules [15]. As our screenings with PVL and HlgCB in C5aR1-expressing cells identified multiple genes in two major PTM pathways, we subsequently assessed the contribution of sulfation and sialylation to *S. aureus* leukocidin susceptibility.

### 2.2. Sulfation of C5aR1 Facilitates both PVL and HlgCB Cytotoxicity.

**Figure 2:**
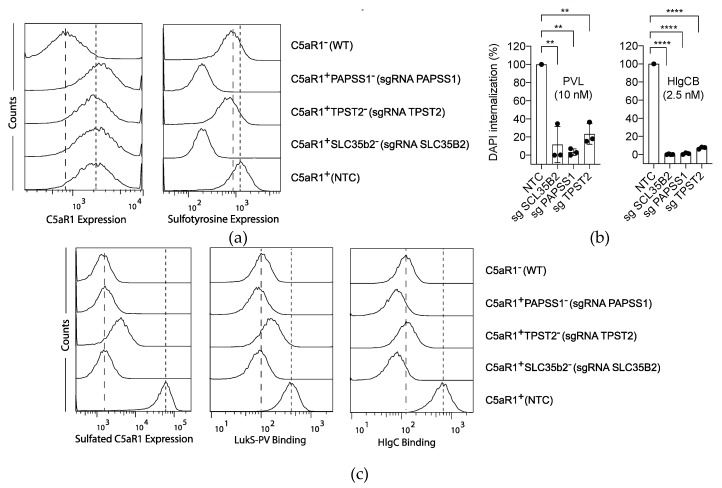
Sulfation of C5aR1 facilitates PVL and HlgCB cytotoxicity. (**a**) Anti-C5aR1 (clone S5/1) and anti-sulfotyrosine antibodies were used to assess the overall expression of C5aR1 and total sulfotyrosine in U937-C5aR1-SpCas9 cell lines transduced with sgRNA for *PAPSS1* (C5aR1^+^ PAPSS1^−^), *TPST2* (C5aR1^+^ TPST2^−^), *SLC35B2* (C5aR1^+^ SLC35b2^−^), non-targeting control sgRNA (NTC, C5aR1^+^), and U937-SpCas9 (WT, C5aR1^−^) cells. Antibody binding was determined by a fluorescent secondary antibody and the fluorescence measured and analyzed by flow cytometry. Dashed line: expression in U937-SpCas9 (WT, C5aR1^−^) cells; dotted line: C5aR1 expression in NTC (C5aR1^+^) U937 cells. Histograms depict representative examples of two independently repeated experiments. (**b**) Validation of the sulfation-pathway hits after genome-wide CRISPR/Cas9 screen for PVL and HlgCB resistance in U937-C5aR1-SpCas9 cells. As a readout for cell permeability, internalization of DAPI was tested at 30 min post-toxin treatment on a monochromator-based microplate reader and expressed in relation to U937-C5aR1-SpCas9 cells transduced with an NTC sgRNA. Mean and s.d. are shown, with *n* = 3. Statistical significance was calculated using ANOVA analysis of variance with Bonferroni posttest correction for multiple comparison. Statistical significance is displayed as ** for *p* < 0.01 and **** for *p* < 0.0001. (**c**) Expression of sulfated C5aR1 in, and binding of polyhistidine-tagged LukS-PV and HlgC to, U937-C5aR1-SpCas9 cell lines transduced with sgRNA for *PAPSS1* (C5aR1^+^ PAPSS1^−^), *TPST2* (C5aR1^+^ TPST2^−^), *SLC35B2* (C5aR1^+^ SLC35b2^−^), non-targeting control sgRNA (NTC, C5aR1^+^), and U937-SpCas9 (WT, C5aR1^−^) cells. Anti-C5aR1 (clone 347214) antibodies were used to assess the expression of sulfated C5aR1. Antibody binding was determined by a fluorescent secondary antibody. Binding of LukS-PV and HlgC was detected with anti-His-FITC antibodies. Fluorescence was measured and analyzed by flow cytometry. Dashed line: expression on and binding to U937-SpCas9 (WT, C5aR1^−^) cells; dotted line: expression on and binding to NTC (C5aR1^+^) U937 cells. Histograms depict representative examples of two independently repeated experiments.

To validate the involvement of *SLC35B2*, *PAPSS1* and *TPST2* in PVL and HlgCB cytotoxicity, single knock-out cells were generated in U937-C5aR1-SpCas9 cells. Single knock-out cells where incubated with different antibodies to assess the expression of specific targets and evaluated by flow cytometry [12]. Individually knocking-out *SLC35B2* or *PAPSS1*, and to an extent *TPST2*, resulted in a decrease of overall cell surface tyrosine sulfation, as determined using an anti-sulfotyrosine antibody (Figure 2a). Consistent results were obtained in single knock-out U937-SpCas9 cells (Figure S1). Lack of tyrosine sulfation did not affect the overall levels of C5aR1 expression, as detected with a sulfation-independent binding anti-C5aR1 antibody (clone S5/1, Figure 2a). Subsequently, C5aR1^+^SLC35b2^−^, C5aR1^+^PAPSS1^−^ and C5aR1^+^TPST2^−^ cells were challenged with PVL and HlgCB. Consistent with the screening results, C5aR1^+^SLC35b2^-^, C5aR1^+^PAPSS1^−^ and C5aR1^+^TPST2^−^ cells showed resistance to pore formation induced by both PVL and HlgCB (Figure 2b). These data identify sulfation as a conserved posttranslational modification for the interaction of both PVL and HlgCB with their target cells.

Binding of LukS-PV, the S-component of PVL, to a synthetic N-terminal C5aR1-peptide was previously shown to be mediated by sulfation of two peptide-tyrosine residues [7]. We hypothesized that knocking-out *SLC35B2*, *PAPSS1* or *TPST2* in U937-C5aR1 cells reduces C5aR1-sulfation and subsequent binding of the S-components LukS-PV and HlgC. During the evaluation of multiple anti-C5aR1 antibodies, we observed a sulfation-dependent binding of the anti-human C5aR1 antibody clone 347214. While the overall level of C5aR1 expression was not affected (Figure 2a), knocking-out *SLC35B2* or *PAPSS1* indeed reduced C5aR1-sulfation as assessed with this sulfation-dependent binding anti-C5aR1 antibody (Figure 2c). In addition, although knocking-out *TPST2* only slightly affected overall tyrosine sulfation (Figure 2a), C5aR1-sulfation was reduced in C5aR1^+^TPST2^−^ cells (Figure 2c). Next, we assessed the binding of LukS-PV and HlgC to these cells. As expected, binding of LukS-PV and HlgC was impaired in C5aR1^+^SLC35b2^−^, C5aR1^+^PAPSS1^−^ and C5aR1^+^TPST2^−^ cells (Figure 2c). Thus, these results show that cellular susceptibility towards both PVL and HlgCB is mediated by a conserved sulfation-dependent binding of LukS-PV and HlgC to C5aR1.

### 2.3. The role of C5aR1 Sialylation in HlgCB- and PVL-Induced Cytotoxicity.

**Figure 3:**
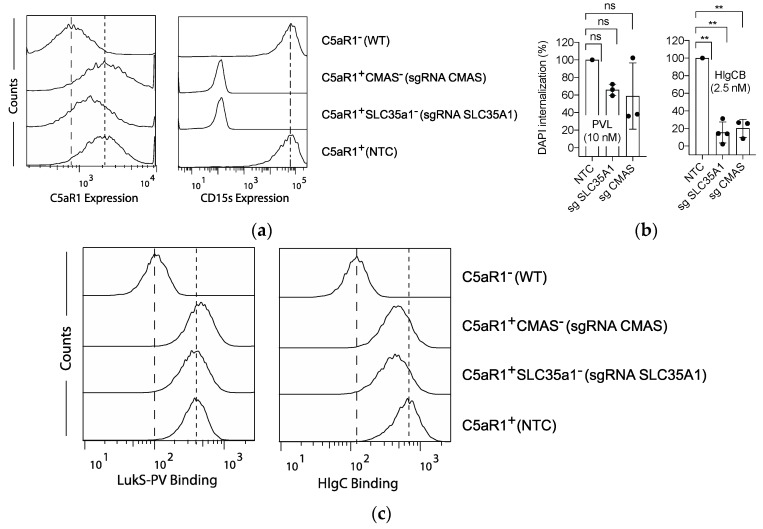
The role of sialylation in HlgCB- and PVL-induced cytotoxicity. (**a**) Anti-C5aR1 and anti-CD15s antibodies were used to assess the expression of C5aR1 and CD15s on U937-C5aR1-SpCas9 cell lines transduced with sgRNA for *CMAS* (C5aR1^+^ CMAS^−^), *SLC35A1* (C5aR1^+^ SLC35a1^−^), non-targeting control sgRNA (NTC, C5aR1^+^), and U937-SpCas9 (WT, C5aR1^−^) cells. Antibody binding was determined by a fluorescent secondary antibody and the fluorescence measured and analyzed by flow cytometry. Dashed line: expression in U937-SpCas9 (WT, C5aR1^−^) cells; dotted line: C5aR1 expression in NTC (C5aR1^+^) U937 cells. Histograms depict representative examples of two independently repeated experiments. (**b**) Validation of the sialylation-pathway hits after genome-wide CRISPR/Cas9 screen for PVL and HlgCB resistance in U937-C5aR1-SpCas9 cells. As a readout for cell permeability, internalization of DAPI was tested at 30 min post-toxin treatment on a monochromator-based microplate reader and expressed in relation to U937-C5aR1-SpCas9 cells transduced with an NTC sgRNA. Mean and s.d. are shown, with *n* = 3. Statistical significance was calculated using ANOVA analysis of variance with Bonferroni posttest correction for multiple comparison. Statistical significance is displayed as ** for *p* < 0.01 and NS for not significant. (**c**) Binding of polyhistidine-tagged LukS-PV and HlgC to U937-C5aR1-SpCas9 cell lines transduced with sgRNA for *CMAS* (C5aR1^+^ CMAS^−^), *SLC35A1* (C5aR1^+^ SLC35a1^−^), non-targeting control sgRNA (NTC, C5aR1^+^) and wild type U937 (C5aR1^−^). Cells were subsequently incubated with anti-His-FITC antibodies and the fluorescence measured and analyzed by flow cytometry. Dashed line: binding to U937-SpCas9 (WT, C5aR1^−^) cells; dotted line: binding to NTC (C5aR1^+^) U937 cells. Histograms depict representative examples of two independently repeated experiments.

In addition to genes involved in tyrosine sulfation, our screening for HlgCB resistance identified genes involved in the sialylation pathway. To confirm these findings, *SLC35A1* and *CMAS* single knock-out cells were generated in U937-C5aR1-SpCas9 cells. The level of sialylated LewisX (CD15s) expression was used as a readout to assess overall cellular sialylation. C5aR1^+^SLC35a1^−^ and C5aR1^+^CMAS^−^ cells showed no binding of anti-Sialyl-LewisX antibodies (Figure 3a), confirming the lack of sialic acid modifications in these cells. Consistent results were obtained in single knock-out U937-SpCas9 cells (Figure S1). The overall C5aR1-expression was mildly impaired in the mutant U937-C5aR1-SpCas9 cell lines (Figure 3a).

Subsequent toxin challenge confirmed a reduced susceptibility to pore formation induced by HlgCB in C5aR1^+^SLC35A1^-^ and C5aR1^+^CMAS^−^ cells (Figure 3b). As *SLC35A1* was also identified in our PVL-resistance screen (yet with a lower enrichment score, Figure 1 and Tables S1 and S2), we challenged C5aR1^+^SLC35a1^−^ and C5aR1^+^CMAS^−^ cells with PVL as well. Knocking-out *SLC35A1* or *CMAS* however reduced cell susceptibility towards PVL only mildly and in a statistically insignificant manner (Figure 3b). Thus, these data identify sialylation of C5aR1 as a PTM necessary for the interaction with HlgCB and, to a lesser extent, PVL.

As with the sulfation pathway, we hypothesized that reduced susceptibility of C5aR1^+^SLC35A1^−^ and C5aR1^+^CMAS^−^ cells to HlgCB and PVL is due to an impaired interaction of the toxin’s S-component with C5aR1. To test this, we determined the binding of HlgC and LukS-PV to C5aR1^+^SLC35a1^−^ and C5aR1^+^CMAS^−^ cells. Although binding of HlgC to C5aR1^+^SLC35a1^−^ and C5aR1^+^CMAS^−^ cells was indeed reduced (Figure 3c), no differences were detected in the binding of LukS-PV (Figure 3c).

Taken together, these results show that sialylation of C5aR1 is a molecular determinant mediating susceptibly to HlgCB more than PVL. For HlgCB, sialylation drives refinement of cytotoxicity by facilitating the binding of the S-component, combined with a modulation of receptor expression levels. For PVL, the limited impact of sialylation on cellular susceptibility is likely mediated by receptor surface expression levels solely.

### 2.4. Sulfation and to a Lesser Extent Sialylation Refine Susceptibility to PVL and HlgCB.

To further assess the extent of the role of *SLC35B2*, *PAPSS1*, *TPST2*, *SLC35A1* and *CMAS* in cellular susceptibility to PVL and HlgCB, mutant cells were incubated with different concentrations of PVL or HlgCB. Pore formation was defined as the time and concentration dependent collective DAPI-internalization (Figure S2). The area under the curve was calculated and subsequently related to U937-C5aR1-SpCas9 cells transduced with a non-targeting control sgRNA [12].

**Figure 4:**
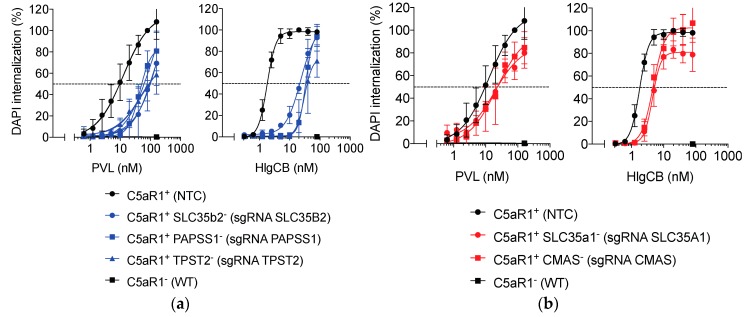
Sulfation and sialylation of C5aR1 refine susceptibility to PVL and HlgCB. Susceptibility of U937-C5aR1-SpCas9 cell lines transduced with sgRNA for (**a**) the sulfation pathway genes *SLC35B2* (C5aR1^+^ SLC35b2^−^), *PAPSS1* (C5aR1^+^ PAPSS1^−^), *TPST2* (C5aR1^+^ TPST2^−^), and (**b**) the sialylation pathway genes *SLC35A1* (C5aR1^+^ SLC35a1^−^), *CMAS* (C5aR1^+^ CMAS^−^), a non-targeting control sgRNA (NTC, C5aR1^+^), and U937-SpCas9 (WT, C5aR1^−^) cells to PVL and HlgCB. As a readout for cell permeability, internalization of DAPI was measured during 30 min post-toxin treatment on a monochromator-based microplate reader and expressed in relation to the area under the curve for NTC sgRNA transduced U937-C5aR1-SpCas9 cells at 80 nM PVL or 20 nM HlgCB.

As previously reported, the expression of C5aR1 was essential for sensitization of cells towards PVL and HlgCB cytotoxicity [7,16]. Results obtained for the individual genes in the sulfation pathway (*SLC35B2*, *PAPSS1*, and *TPST2*) were similar, as were those for the genes in the sialylation pathway (*SLC35A1* and *CMAS*). The absence of cellular sulfation resulted in a ~5-fold increase in the half-maximum effective concentration (EC50) for PVL and a ~15-fold increase in the EC50 for HlgCB (Figure 4a and Table 1). The increase in the EC50 in cells lacking sialylation was limited, with a ~2.5-fold shift for HlgCB and a statistically insignificant trend towards a ~1.5-fold shift for PVL (Figure 4b and Table 1).

These findings show that, although sialylation and sulfation of C5aR1 are not essential for PVL and HlgCB cytotoxicity at high toxin concentrations, these PTM pathways further refine toxin susceptibility of human phagocytic cells. For the C5aR1-targeting leukocidins PVL and HlgCB, the impact of sulfation is more extensive when compared to sialylation.

### 2.5. Sialyation and to a Lesser Extent Sulfation Refine Susceptibility to LukED and HlgAB.

**Figure 5:**
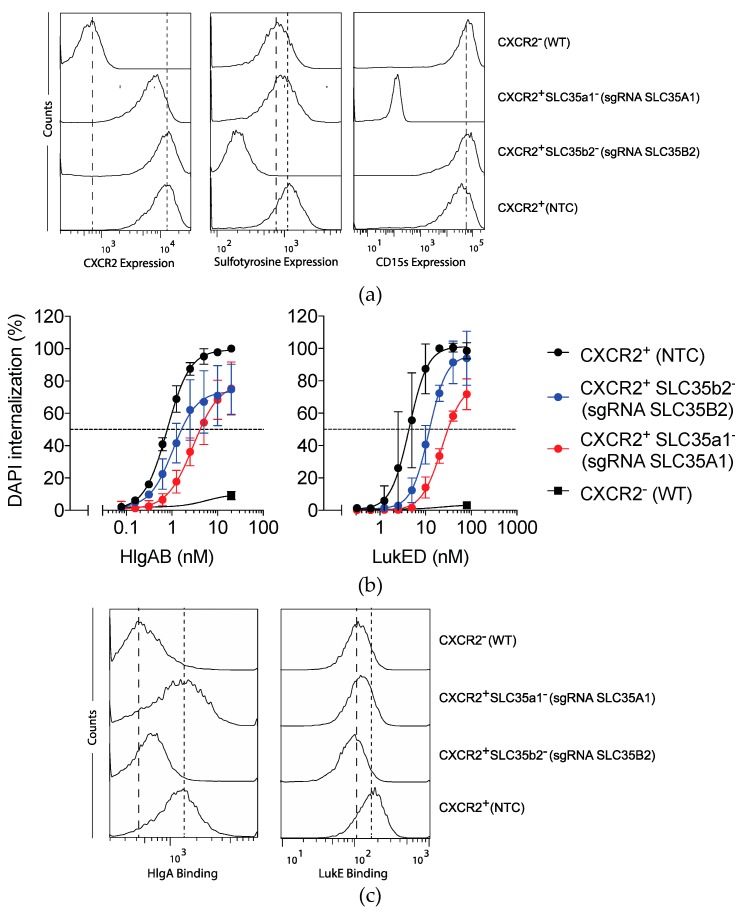
Sialylation and sulfation of CXCR2 refine susceptibility to LukED and HlgAB. (**a**) Anti-CXCR2, anti-sulfotyrosine and anti-CD15s antibodies were used to assess the expression of CXCR2, total sulfotyrosine, and CD15s on U937-CXCR2-SpCas9 cell lines transduced with sgRNA for *SLC35A1* (CXCR2^+^ SLC35a1^−^), *SLC35B2* (CXCR2^+^ SLC35b2^−^), non-targeting control sgRNA (NTC, CXCR2^+^), and U937-SpCas9 (WT, CXCR2^−^) cells. Antibody binding was determined by a fluorescent secondary antibody and the fluorescence measured and analyzed by flow cytometry. Dashed line: expression in U937-SpCas9 (WT, CXCR2^−^) cells; dotted line: expression in NTC (CXCR2^+^) U937 cells. Histograms depict representative examples of two independently repeated experiments. (**b**) Susceptibility of U937-CXCR2-SpCas9 cell lines transduced with sgRNA for *SLC35B2* (CXCR2^+^ SLC35b2^−^), *SLC35A1* (CXCR2^+^ SLC35a1^−^), non-targeting control sgRNA (NTC, CXCR2^+^), and U937-SpCas9 (WT, CXCR2^−^) cells to HlgAB and LukED. As a readout for cell permeability, internalization of DAPI was measured during 30 min post-toxin treatment on a monochromator-based microplate reader and expressed in relation to the area under the curve for NTC sgRNA transduced U937-CXCR2-SpCas9 cells at 20 nM HlgAB or LukED. (**c**) Binding of Alexa Fluor 647 maleimide-based labeled HlgA and polyhistidine-tagged LukE to U937-CXCR2-SpCas9 cell lines transduced with sgRNA for *SLC35B2* (CXCR2^+^ SLC35b2^−^), *SLC35A1* (CXCR2^+^ SLC35a1^−^), non-targeting control sgRNA (NTC, CXCR2^+^), and U937-SpCas9 (WT, CXCR2^−^) cells. Cells were subsequently incubated with anti-histidine-FITC secondary antibodies and/or the fluorescence was directly measured and analyzed by flow cytometry. Dashed line: binding to U937-SpCas9 (WT, CXCR2^−^) cells; dotted line: binding to NTC (CXCR2^+^) U937 cells. Histograms depict representative examples of two independently repeated experiments.

We hypothesized that PTMs of host receptors not only play a role in the interaction of C5aR1-interacting toxins, but also in the interaction of other leukocidins with their GPCR-targets. To study the role of sulfation and sialylation in LukED and HlgAB receptor interactions, we engineered specific gene deletants in U937 cells ectopically expressing their shared cell surface target CXCR2 (U937-CXCR2-SpCas9). As representatives of the sialylation and sulfation pathways, *SLC35B2* and *SLC35A1* were selected for gene editing. As with C5aR1^+^SLC35a1^−^ cells, expression of CXCR2 on CXCR2^+^SLC35a1^−^ cells was mildly impaired while CXCR2 expression on CXCR2^+^SLC35b2^−^ cells was unaffected (Figure 5a). CXCR2^+^SLC35b2^−^ cells lacked detectable levels of sulfation while CXCR2^+^SLC35a1^−^ cells were devoid of sialylation (Figure 5a).

Next, CXCR2^+^SLC35a1^−^ and CXCR2^+^SLC35b2^−^ cells were challenged with a concentration range of HlgAB or LukED. In line with previous reports, CXCR2-expression was essential for sensitization of cells towards both HlgAB and LukED cytotoxicity (Figure 5b) [8,9]. Opposite to our observations with C5aR1, CXCR2^+^SLC35a1^−^ cells were ~4 and ~5 fold less sensitive to both HlgAB and LukED, respectively (Figure 5b and Table 2) while CXCR2^+^SLC35b2^−^ cells showed only a minor trend towards a decreased sensitivity for HlgAB and LukED (Figure 5b and Table 2). Thus, for the CXCR1-targeting leukocidins HlgAB and LukED, the impact of sialylation is more extensive when compared to sulfation.

As with C5aR1, we subsequently investigated if the observed PTM-dependent resistance in CXCR2-expressing cells could be correlated to a reduced binding of HlgA or LukE, the respective S-components of HlgAB and LukED. Reminiscent of C5aR1-expressing cells and LukS-PV and HlgC, binding of HlgA and LukE was reduced in CXCR2^+^SLC35b2^−^ cells. Although CXCR2^+^SLC35a1^−^ cells showed a reduced susceptibility to pore formation when challenged with HlgAB or LukED, only a reduced binding of LukE, but not HlgA, could be detected in CXCR2^+^SLC35a1^−^ cells (Figure 5c). This apparent discrepancy may be attributed to the facts that 1) the overall signals for HlgA and LukE-binding in CXCR2^+^ cells are low (Figure 5c), and 2) different strategies were applied to detect binding of HlgA and LukE, respectively.

Collectively, these results show that in contrast to C5aR1, susceptibility to leukocidins in CXCR2 expressing cells is mostly driven by sialylation instead of sulfation. For LukED, sialylation drives cytotoxicity by facilitating the binding of the S-component, combined with a modulation of receptor expression levels. For HlgAB, the impact of sialylation on cellular susceptibility is likely mediated by receptor surface expression levels only. Sulfation has a limited impact on HlgAB and LukED cytotoxicity of CXCR2-expressing cells, by affecting the binding of the leucocidin S-components. When taken together, our genome-wide screenings highlight a conserved yet divergent role of PTMs in refining susceptibility of human phagocytic cells to the staphylococcal leukocidins.

## 3. Discussion

We used a genome-wide CRISPR/Cas9 based approach to screen for cellular factors involved in susceptibility towards GPCR-targeting leukocidins, and identify two PTM pathways (sulfation and sialylation) driving GPCR-mediated susceptibility of phagocytes to leukocidins. Post-translational enzymatic reactions modify specific protein backbones and sidechains, thereby modulating protein function [17]. PTMs of GPCRs are important for regulating structure, function and association of the receptors with their natural ligands [18,19]. In addition, PTM moieties on GPCRs have also been implied in the interaction with different pathogens [20,21,22]. Hemolytic activity of the *Streptococcus pneumoniae* cytolysin Ply was recently shown to be mediated by its interaction with sialylated Lewis X histo-blood group antigen [22]. Neutralization of HlgCB cytotoxicity by pre-incubation with the ganglioside GM1, a sialylated oligosaccharide [23] further suggests a role for sialylation in leukocidin toxicity. Using a synthetic N-terminal C5aR1 peptide, it was previously proposed that tyrosine-sulfation facilitates binding of LukS-PV [7]. However, it has remained undetermined to what extent GPCR-sulfation contributes to pore formation. In addition, it is unknown if PTMs define a conserved signature for the interaction between the leukocidins and their respective GPCRs.

Disruption of the sulfation pathway results in a reduced S-component binding to GPCR-expressing U937 cells, supporting the notion that GPCR-sulfation enhances target cell susceptibility to leukocidins by strictly facilitating binding of the S-components [7]. Disruption of the sialylation pathway however likely results in a combination of a reduced binding as well as a compromised overall receptor expression. The observed reduction in binding of the S-components to the sialylation-mutant cell lines is limited (if reduced at all), and no monoclonal antibodies are currently available for specific detection of C5aR1 or CXCR2 in a sialylation-dependent manner. Therefore, it cannot be excluded that the interaction of the toxins with CD45 (the receptor for LukF-PV [12]) or elusive receptors of the other leukocidin F-components is mediated by sialylation as well.

C5aR1-expressing sulfation-deficient cells display a reduced susceptibility towards both PVL and HlgCB. In addition, our data indicate that posttranslational decoration of C5aR1-expressing cells with sialylation-moieties facilitates PVL and HlgCB cytotoxicity. Compared to sulfation, however, the contribution of sialylation to C5aR1-interacting leukocidin susceptibility is limited. Overall, this screening identifies sulfation-mediated receptor-employment as a major and conserved feature for C5aR1-interacting leukocidins. In contrast to C5aR1, sulfation of CXCR2 expressing cells does not have a major impact on LukED and HlgAB cytotoxicity. Although the CXCR2 N-terminus contains two tyrosines, it lacks acidic sulfation modification motifs that mediate TPST2 activity. DARC, the erythrocyte receptor for HlgAB and LukED and a close homologue of CXCR2, has been suggested to interact with both leukocidins in an N-terminal sulfated tyrosine dependent manner [11]. In contrast to CXCR2, the DARC N-terminus does contain tyrosine sulfation determinants. Likely, CXCR2 is sulfated with less efficiency [24,25]. This indicates a divergent role for GPCR-sulfation in leukocidin susceptibility, in which LukED and HlgAB interact sulfation-dependent in a receptor-specific manner. Opposed to our findings in C5aR1, our data identify sialylation rather than sulfation as the major PTM motif facilitating cytotoxicity of CXCR2-targeting leukocidins.

Multiple extracellular domains of the leukocidin receptors are involved during pore formation, and conformational changes of the GPCRs are likely to occur in the process of binding, hetero-oligomerization, and pore formation [16]. In addition to directly enhancing the binding of leukocidins and regulating overall receptor expression levels, PTMs possibly also affect the GPCR structures and their conformational plasticity [19]. These observations identify PTMs of GPCRs as potential targets for pharmacological interference during infection. Editing of individual genes encoding proteins involved in the sialylation (*CMAS*, *SLC35A1*) or sulfation pathway (*SLC35B2*, *PAPSS1*, *TPST2*) all resulted in disruption of overall sialylation or sulfation in GPCR-expressing cells, implying that the respective genes are part of a non-redundant and sequential pathway. To the best of our knowledge, no pharmacological compounds targeting the tyrosine sulfation pathway are currently being studied. As *TPST1* and *TPST2* double knockout mice die in the early postnatal phase [26], pharmaceutical interference with the sulfation pathway will likely be challenging due to the essentiality of this pathway in eukaryotic cell homeostasis. Although competitive small-molecule inhibitors of sialyltranferases are being developed, toxic side effects of currently available compounds will limit their applicability in humans on short term [27,28].

The necessity of the seemingly redundant range of phagocyte-targeting toxins produced by *S. aureus* is incompletely understood [5]. Recent studies have revealed similarities and differences in the interactions between the leukocidins and their respective GPCRs [8,11,16]. The differences in the impact of PTM moieties on leukocidin toxicity highlight an additional layer of refinement and divergence of the staphylococcal host-pathogen interaction. These differences further challenge the apparent functional redundancy of the leukocidins, and enhance our understanding of their cellular tropism and species specificity.

Expression of the leukocidin S-component receptors is essential for pore formation [5]. The PTM pathways identified in this study serve as refining mechanisms that further enhance susceptibility of leukocidin-receptor expressing cells to pore formation. PTMs display a wide heterogeneity in terms of cell type and tissue specificity [19,29]. While *S. aureus* is capable of causing a plethora of infections, its tissue tropism is poorly understood. Cell type and organ specific variations in sulfation and sialylation, contributing to local cellular susceptibility towards the leukocidins, may possibly contribute to organ specific *S. aureus* infections.

The mechanisms for the predisposition of otherwise healthy individuals to severe infections with *S. aureus* are poorly understood [1,30,31,32]. Variations in the genes encoding PTM pathways could provide insight into observed differences in susceptibility of humans to severe infections with *S. aureus*. It remains to be established if specific variants of the sialylation and sulfation pathway genes alter susceptibility to *S. aureus* infections in humans. The combination of a preceding Influenza-virus infection and a subsequent *S. aureus* pneumonia is well known for its poor outcome of disease [30,33,34,35]. The interaction of Influenza-virus neuraminidase with sialic acids in the lungs vs. the sialylation-mediated employment of GPCRs by the staphylococcal leukocidins deserves further study.

## 4. Materials and Methods

### 4.1. Cell Lines and Constructs

U937 human monocytic cells were obtained from ATCC (American Type Culture Collection) and cultured in RPMI supplemented with penicillin/streptomycin and 10% fetal calf serum. Cell lines were constructed as previously described [12]. Briefly, to sensitize the cells to PVL and HlgCB or LukED and HlgCB, human C5aR1 (CD88; NM_001736) and CXCR2 (CD182; NM_001168298.1) were stably expressed in U937 cells using a lentiviral expression system (U937-C5aR1, U937-CXCR2 cells). We cloned the human *C5AR1* and human *CXCR2* cDNA in a dual promoter lentiviral vector (BIC-PGKZeo- T2a-mAmetrine; RP172), derived from no.2025.pCCLsin.PPT.pA.CTE.4x-scrT. eGFP.mCMV.hPGK.NG-FR.pre as described elsewhere [36]. The transfection of 293T cells with the C5aR1 and CXCR2 lentiviral expression systems, and subsequent transduction of U937-cells were performed as previously described [12]. To allow screening, a codon-optimized nuclear-localized *S. pyogenes* cas9 gene was subsequently transduced in U937-C5aR1 as described elsewhere [12]. The genome-wide sgRNA CRISPR/Cas9 library was designed as previously described [12]. The following sgRNA sequences were cloned in sgLenti to generate SLC35B2 (fw- 5′ TTGGACAGGCTGGCAAAG-GAGTAC 3′, rv 3′ AAACGTACTCCTTTGCCAGCCTGT 5′), PAPSS1 (fw- 5′ TTGGGCAAGTTGTG-GAACTTCTAC 3′, rv- 3′ AAACGTAGAAGTTCCACAACTTGC 5′), TPST2 (fw- 5′ TTGGGGCCCG-CGTGCTCTGCAACA 3′, rv- 3′ AAACTGTTGCAGAGCACGCGGG- CC 5′), SLC35A1 (fw- 5′ TTG-GACATACAAGAAGAGTACCCA 3′, rv- 3′ AAACTGGGTACTCTT- CTTGTATGT 5′) and CMAS (fw- 5′ TTGGGAGAATGTGGCCAAACAATT 3′, rv- 3′ AAACAATT- GTTTGGCCACATTCTC 5′) knockout cell lines. U937-SpCas9, U937-C5aR1-SpCas9 or U937-CXCR2-SpCas9 cells were subsequently transduced with the sgRNA-expression viruses and selected to purity by puromycin treatment (2 μg/mL) to enrich for knocked-out cells.

### 4.2. Genome-Wide CRISPR/Cas9 Library Screen in U937-C5aR1 Cells

The genome-wide CRISPR/Cas9 screen for leukocidin resistance was performed as described elsewhere [12]. Briefly, cells transduced with the CRISPR sgRNA library were selected to purity with puromycin (2 μg/mL) initiated at two days post transduction. Twelve days post transduction, 2 × 10^8^ cells were incubated with 15 nM PVL or 15 nM HlgCB for 30 min at 37 °C, which resulted in depletion of >99.5% of the cells. Cells were washed to remove the toxin and allowed to recover in complete RPMI for 15 days to enrich for viable cells. Genomic DNA was isolated and sgRNA inserts were subsequently PCR amplified for 16 cycles with primers 5′ GGCTTGGATTTCTATAACTTCGT- ATAGCA 3′ and 5′ CGGGGACTGTGGGCGATGTG 3′ using the Titanium Taq PCR kit (Clontech, Göteborg, Sweden). The PCR products were pooled and amplified using primers containing Illumina adapter sequences and a unique index. PCR products were subsequently pooled in equimolar ratios and subjected to deep-sequencing using the Illumina NextSeq500 platform. Sequences were aligned to the sgRNA library by using Bowtie2 (Johns Hopkins University, Baltimore, MA, USA; PMID: 22388286) and the counts per sgRNA were calculated. We used the MaGeCk package (Dana-Farber Cancer Institute, Boston, MA, USA; PMID: 25476604; available from https://sourceforge.net/projects/ mageck/) as a computational tool to identify genes significantly enriched in the screens by comparing sgRNA read counts of control cells versus PVL- and HlgCB incubated cells. Raw data obtained from the PVL resistance screen were previously reported [12].

### 4.3. Recombinant Protein Production and Cell Permeability Assays

Polyhistidine-tagged LukS-PV, LukF-PV, HlgC, HlgA, HlgB, LukE, LukD and Alexa Fluor 647 maleimide-based labeled HlgA used during this study were cloned and expressed as described elsewhere [7,8,37,38]. For permeability assays, each U937 cell line (1 × 10^7^ cells per ml in RPMI/HSA) was exposed to recombinant toxins and measured for 30 min at 37 °C in a monochromator-based microplate reader (FLUOstar Omega, BMG Labtech, Ortenberg, Germany) in the presence of 2,5 μg/mL 4′,6-diamidino-2-phenylindole (DAPI, Molecular Probes/Thermo Fisher, Landsmeer, The Netherlands) to determine pore-formation [12]. As PVL, HlgCB, HlgAB and LukED are two-component toxins, equimolar concentrations of polyhistidine-tagged S- and F-components were used. Pore formation was defined as the concentration dependent collective DAPI-internalization during the course of 30 min. The area under the curve (AUC), corrected for toxin-independent DAPI-internalization, was calculated and subsequently related to the maximum AUC obtained in U937-C5aR1-SpCas9 or U937-CXCR2-SpCas9 cells transduced with a non-targeting control sgRNA that were treated with the highest respective toxin concentration (Figure S2) [12]. Cell lines lacking expression of C5aR1 or CXCR2 (U937-SpCas9 cell lines) are resistant to DAPI-internalization upon toxin-exposure.

### 4.4. Determination of Receptor Expression Levels and Binding Assays

Receptor expression levels were determined as described elsewhere [8]. Briefly, single U937 cell suspensions were stained with mouse anti-human C5aR1 (Sulfation independent clone S5/1, AbD Serotec, Kidlington, UK), mouse anti-human C5aR1 (Sulfation dependent clone 347214, R&D Systems, Abingdon, UK) mouse anti-human CXCR2 (clone 6D499, Abnova, Heidelberg, Germany), mouse-anti-sulfotyrosine (Clone Sulfo-1C-A2, EMD Millipore, Burlington, MA, USA), mouse-anti-CD15s (Clone CLSLEX1, BD Pharmingen, Erembodegem, Belgium), followed by APC-conjugated goat-anti-mouse antibody (Jackson Immunoresearch, Westgrove, PA, USA). Samples were subsequently measured using flow cytometry. For the S-component binding studies on U937 cells, cells were incubated with 10 μg/mL polyhistidine-tagged LukS-PV or HlgC for 30 min on ice followed by anti-his-FITC (LifeSpan BioSciences, Seattle, WA, USA), or 10 μg/mL polyhistidine-tagged LukE for 20 min at 20 °C followed by anti-his-FITC (LifeSpan BioSciences, Seattle, WA, USA), or with 10 μg/mL Alexa Fluor 647 maleimide-based labeled HlgA [38] for 20 min at 20 °C. Cells were subsequently measured using flow cytometry.

### 4.5. Statistical Analyses

Calculations of the area under the curves, calculations of half-maximal effective lytic concentrations using linear regression analyses, and all statistical analyses were performed using Prism 7.0 (GraphPad Software, San Diego, CA, USA). Statistical significance was calculated using ANOVA analysis of variance with Bonferroni posttest correction for multiple comparison where appropriate. Four-parametric non-linear regression analyses were performed to obtain half-maximum effective concentrations (EC50s). Flow cytometric data were analyzed with FlowJo (Tree Star Software, Erembodegem, Belgium).

## Figures and Tables

**Table 1:** Sulfation and sialylation of C5aR1 refine susceptibility to PVL and HlgCB. Half-maximum effective concentrations (EC50s) of U937-C5aR1-SpCas9 cells transduced with a non-targeting control sgRNA (NTC, C5aR1^+^) and the EC50 and fold increase in U937-C5aR1-SpCas9 cell lines transduced with the sgRNA *SLC35B2* (C5aR1^+^ SLC35b2^−^), *PAPSS1* (C5aR1^+^ PAPSS1^−^), *TPST2* (C5aR1^+^ TPST2^−^), *SLC35A1* (C5aR1^+^ SLC35a1^−^) and *CMAS* (C5aR1^+^ CMAS^−^) after exposure to PVL or HlgCB. EC50 values were calculated using four-parametric non-linear regression analyses. Fold increased EC50 values are expressed in relation to the EC50 of the NTC, with their corresponding 95% confidence interval.

sgRNA		PVL			HlgCB	
	EC50 (nM)	95% Conf. Interval (nM)	Fold Increased EC50	EC50 (nM)	95% Conf. Interval (nM)	Fold Increased EC50
NTC	12.9	9.1–21.5		1.8	1.7–1.9	
*SLC35B2*	123.1	38.1–infin	9.5	30.3	23.1–50.6	16.8
*PAPSS1*	44.6	35.7–63.4	3.5	33.3	30.6–36.3	18.5
*TPST2*	42.5	22.2–infin	3.3	23.1	18.6–34.3	12.8
*SLC35A1*	18.5	11.5–67.4	1.4	4.5	3.9–5.1	2.5
*CMAS*	19.4	11.3–79.6	1.5	4.7	4.0–5.6	2.6

**Table 2:** Sulfation and sialylation of CXCR2 refine susceptibility to LukED and HlgAB. Half-maximum effective concentrations (EC50s) of U937-CXCR2-SpCas9 cells transduced with a non-targeting control sgRNA (NTC, CXCR2^+^) and the EC50 and fold increase in U937-CXCR2-SpCas9 cell lines transduced with the sgRNA *SLC35B2* (CXCR2^+^ SLC35b2^−^) and *SLC35A1* (CXCR2^+^ SLC35a1^−^) after exposure to HlgAB or LukED. EC50 values were calculated using four-parametric non-linear regression analyses. Fold increased EC50 values are expressed in relation to the EC50 of the NTC, with their corresponding 95% confidence interval.

sgRNA		HlgAB			LukED	
	EC50 (nM)	95% Conf. Interval (nM)	Fold Increased EC50	EC50 (nM)	95% Conf. Interval (nM)	Fold Increased EC50
NTC	0.8	0.7–0.9		4.4	3.4–5.7	
*SLC35B2*	1.1	0.7–1.7	1.4	11.8	10.1–14.0	2.7
*SLC35A1*	2.9	2.1–5.1	3.6	22.7	19.8–27.3	5.2

## Supplementary Materials

The following are available online at https://www.mdpi.com/2072-6651/12/2/106/s1: Table S1 (Related to Figure 1): Screening results for resistance to PVL toxicity - CRISPR/Cas9 library screen for PVL resistance set up in U937-C5aR1-SpCas9 cells, selected after exposure to PVL. Table S2 (Related to Figure 1): Screening results for resistance to HlgCB toxicity - CRISPR/Cas9 library screen for HlgCB resistance set up in U937-C5aR1-SpCas9 cells, selected after exposure to HlgCB. Figure S1 (Related to Figure 2 and Figure 3): Sulfation and sialylation in U937-SpCas9 cell lines. Figure S2 (Related to Figure 4 and Materials and methods): Time and concentration dependent DAPI-internalization.
