# Supplementary material for: Host–Receptor Post-Translational Modifications Refine Staphylococcal Leukocidin Cytotoxicity
Source: Toxins (Basel). 2020 Feb 6;12(2):106. doi: 10.3390/toxins12020106 (PMC7076806; doi:10.3390/toxins12020106)
Supplement: Supplementary file 1 [file toxins-12-00106-s001.zip › toxins-705920-SI.docx]

Supplementary Matherials: Host–Receptor Post-Translational Modifications Refine Staphylococcal Leukocidin Cytotoxicity

Angelino T. Tromp, Michiel Van Gent, Joris P. Jansen, Lisette M. Scheepmaker, Anneroos Velthuizen, Carla J.C. De Haas, Kok P.M. Van Kessel, Bart W. Bardoel, Michael Boettcher, Michael T. McManus, Jos A.G. Van Strijp, Robert Jan Lebbink, Pieter-Jan A. Haas and András N. Spaan

|   (**a**) |   (**b**) |
| --- | --- |

**Figure S1.** Sulfation and sialylation in U937-SpCas9 cell lines. (**a**) Anti-sulfotyrosine antibodies were used to assess the expression of total sulfotyrosine on U937-SpCas9 cell lines transduced with sgRNA for *PAPSS1* (C5aR1^-^ PAPSS1^-^), *TPST2* (C5aR1^-^ TPST2^-^), *SLC35B2* (C5aR1^-^ SLC35b2^-^), and U937-SpCas9 (WT, C5aR1^-^) cells. Antibody binding was determined by a fluorescent secondary antibody and the fluorescence measured and analysed by flow cytometry. Dashed line: expression in U937-SpCas9 (WT, C5aR1^-^) cells. Histograms depict representative examples of two independently repeated experiments. (**b**) Anti-CD15s antibodies were used to assess the expression of CD15s on U937-SpCas9 cell lines transduced with sgRNA for CMAS (C5aR1^-^ CMAS^-^), SLC35A1 (C5aR1^-^ SLC35a1^-^), and U937-SpCas9 (WT, C5aR1^-^) cells. Antibody binding was determined by a fluorescent secondary antibody and the fluorescence measured and analyzed by flow cytometry. Dashed line: expression in U937-SpCas9 (WT, C5aR1^-^) cells. Histograms depict representative examples of two independently repeated experiments.

**.**

**Figure S2.** Time and concentration dependent DAPI-internalization. Representative example of the areas under the curve for DAPI-internalization used as a readout for cell permeability, as obtained following treatment of U937-C5aR1-SpCas9 cells with two-fold diluted concentrations of PVL (light: 160 nM, dark: 0 nM). Internalization of DAPI was measured during 30 minutes post toxin-treatment on a monochromator-based microplate reader.
